# The Role of G Protein-Coupled Receptors in the Regulation of Orthopaedic Diseases by Gut Microbiota

**DOI:** 10.3390/nu17101702

**Published:** 2025-05-16

**Authors:** Peng Sun, Jinchao Liu, Guannan Chen, Yilan Guo

**Affiliations:** 1 College of Physical Education and Health, East China Normal University, Shanghai 200241, China; 2 The Key Laboratory of Adolescent Health Assessment and Exercise Intervention of the Ministry of Education, East China Normal University, Shanghai 200241, China

**Keywords:** gut microbiota, GPCRS, orthopaedic diseases, metabolites, exercise, diet

## Abstract

Exercise and diet modulate the gut microbiota, which is involved in the regulation of orthopaedic diseases and synthesises a wide range of metabolites that modulate cellular function and play an important role in bone development, remodelling and disease. G protein-coupled receptors (GPCRs), the largest family of transmembrane receptors in the human body, interact with gut microbial metabolites to regulate relevant pathological processes. This paper provides a review of different dietary and exercise effects on the pathogenic gut microbiota and their metabolites associated with GPCRs in orthopaedic diseases. RESULTS: Generally, metabolites produced by gut microbiota contribute to the maintenance of bone health by activating the corresponding GPCRs, which are involved in bone metabolism, regulation of immune response, and maintenance of gut flora homeostasis. Exercise and diet can influence gut microbiota, and an imbalance in gut microbiota homeostasis can trigger a series of adverse immune and metabolic responses by affecting GPCR function, ultimately leading to the onset and progression of various orthopaedic diseases. Understanding these relationships is crucial for elucidating the pathogenesis of orthopaedic diseases and developing personalised probiotic-based therapeutic strategies. In the future, we should further explore how to prevent and treat orthopaedic diseases through GPCR-based modulation of gut microbes and their interactions. The development of substances that precisely modulate gut microbes through different exercises and diets will provide more effective interventions to improve bone health in patients.

## 1. Introduction

Specific orthopaedic diseases such as osteoarthritis, rheumatoid arthritis, and osteoporosis have a rising disease burden globally [1,2,3,4,5]. The worldwide prevalence of orthopaedic diseases and the challenges they pose to public health systems are highlighted by this rising trend.

The gut microbiota is recognised as a key regulatory axis of overall metabolism and is involved in regulating the development of orthopaedic diseases [6]. Gut microbial imbalances are accompanied by host metabolic abnormalities, and pathologic processes are primarily influenced by specific gut microbes through altered metabolite levels [7]. In the gut microbiota, an increase in *Bifidobacteria*, *Lactobacillus*, and other bacteria promotes the synthesis of short-chain fatty acids [8,9]. Bile acids, lactic acid, and succinic acid are key regulators of host health [10]. Acting as signalling molecules, they interact with cellular pathways and receptors, notably GPCRs which directly contribute to the value-added and differentiation of osteoblasts, thus promoting fracture healing [11]. In orthopaedic fractures, alterations in the bone microenvironment, including changes in immune factors, abnormal chemotaxis, and differentiation of mesenchymal stem cells (MSCs), affect fracture healing via the immune pathway [12].

GPCRs, a diverse family of cell-surface receptors, is pivotal for sensing gut microbiota metabolites [13]. They participate in numerous metabolic pathways, including nutrient synthesis, transport, and storage, playing a crucial role in the development and progression of orthopaedic diseases [14]. GPCRs remain one of the most important drug targets, accounting for approximately 34% of FDA-approved drugs, and variant drugs can modulate GPCR signalling without blocking endogenous ligand binding, thereby maintaining beneficial signalling at the receptor and preventing unwanted side effects [10]. GPCR pharmacogenomics provides the means for personalised medicine. Through the study of GPCR, it is possible to develop personalised treatment plans based on the genomic information of patients with orthopaedic diseases through different exercises and diets [15].

In summary, GPCRs has a complex role in relation to the gut microbiota. This includes sensing microbial metabolites, regulating host metabolism, and influencing behaviour and disease progression. The gut microbiota shows substantial individual differences, which are highly relevant to the development and treatment of orthopaedic diseases. GPCRs have different functions and interactions with the gut microbiota that hold great promise for personalised therapeutic approaches. Understanding these mechanisms is critical for the development of targeted therapies that modulate the gut microbiota–GPCR axis for the treatment of orthopaedic diseases.

## 2. The Relationship Between Gut Microbiota and Orthopaedic Disease

In a healthy state, the gut microbiota, a diverse community of microbes in the intestine, maintains a dynamic balance with the host, keeping a stable ratio between beneficial and harmful bacteria. But orthopaedic diseases like osteoporosis and osteoarthritis can disrupt this balance, changing the microbiota’s composition and harming host health [16]. The gut microbes are involved in digestion, metabolism, immune system regulation, and pathogen defence [17,18,19].

### 2.1. Gut Microbiota and Rheumatoid Arthritis

Rheumatoid arthritis (RA), a chronic autoimmune disease, may have its pathogenesis affected by gut microbiota imbalances that can cause autoimmune responses and inflammation. Comparing RA patients and healthy controls shows differences in gut microbiota diversity. Although the α-diversity in RA patients does not change significantly, β-diversity analysis reveals a notable change in the gut microbiota composition [20]. Dysbiosis of specific bacterial lineages and alterations in gut microbiota metabolism led to changes in the host immune profile that contribute to rheumatoid arthritis [21]. Recent studies revealed that the colonisation of *Bacteroides fragilis* is associated with enhanced activity of regulatory T cells (Tregs), which may alleviate autoimmune disease [22,23]. Studies show specific antibiotics can relieve joint swelling, pain, and tenderness in RA patients. In a small-scale clinical trial, RA patients took a combination of gut microbiota-targeting antibiotics and conventional antirheumatic drugs. Post-treatment, patients had lower joint swelling indices, pain scores, and shorter morning stiffness duration [24]. This is likely due to antibiotics modulating the gut microbiota, which affects the immune system’s inflammatory response in the joints.

Multiple research teams used high-throughput sequencing to analyse the gut microbiota of RA patients and healthy controls. Results show that in RA patients, the abundance of pathogenic bacteria like *Prevotella* is higher, and the reduced amount of *Bacteroidetes* may trigger RA through molecular mimicry [25,26]. Granfors et al. (1983) [27] found that the antibody titres against *Yersinia* spp. and *Salmonella* spp. were higher in RA patients’ serum and joint fluids. Their study, using ELISA to measure antibody levels, suggested the gut microbiota might cause disease through antigenic cross-reactivity [27]. Levels of *Prevotella copri* and *Lactobacillus* are increased, while those of *Bacteroidetes*, *Bifidobacteria* and *Eubacterium rectale* are decreased, at an early stage. Abundance of *Lactobacillus salivarius*, *Collinsella*, and *Akkermansia* is increased, while that of *Haemophilus* spp. is decreased, in the active RA phase [26]. A study has shown that *Collinsella aerofaciens*, a commensal gut bacterium found to be overrepresented in RA patients, reduces the expression of tight junction proteins in human intestinal epithelial cells and increases disease incidence in HLA-DQ8 transgenic mice subjected to collagen-induced arthritis (CIA) by disrupting the integrity of the intestinal mucosa [28]. Gut microbiota imbalance disrupts the barrier function, causing overproduction of LPS and TMAO (Trimethylamine *N*-oxide). These substances prompt the body to secrete inflammatory cytokines like TNF-α, IL-1β, and osteoclastogenic differentiation factors. The latter act on osteoclast precursor cells, promoting differentiation and activating the NF-κB signalling pathway [29]. In rheumatoid arthritis, NF-κB activation drives osteoclast differentiation and activation. This increases osteoclast number and activity, accelerating bone resorption and leading to joint bone destruction, which is a key cause of joint damage in RA patients [30].

In summary, β-diversity analysis of the gut microbiota in RA patients revealed significant differences. *Collinsella*, *Akkermansia*, *Prevotella*, *Yersinia*, and *Salmonella* are potentially key pathogenic microbiota in RA.

### 2.2. Gut Microbiota and Osteoarthritis

Osteoarthritis (OA) is characterised by articular cartilage degeneration, and its pathogenesis is complex, involving genetic, environmental, and lifestyle factors. Gut microbial diversity differs between OA patients and healthy controls and is significantly lower in those with severe knee OA [31].

Research shows gut microbiota composition and diversity change in OA patients, with increased abundance of *Streptococcus* spp., *Cholera* spp., and *Desulfovibrio* spp. [32,33]. In the STR/ort mouse model, chronic antibiotic treatment from weaning slowed OA progression [34]. OA patients have a higher proportion of Gram-negative bacteria, which may trigger joint innate immune responses via TLR-4, causing joint degeneration [35]. Cartilage in OA patients has changes in opportunistic pathogenic bacteria, like reduced DNA diversity and enhanced LPS-biosynthesis pathways. In a guinea pig model of spontaneous OA, oral *Bifidobacterium longum* CBi0703 treatment protected joints by reducing cartilage damage and degradation markers [32,33,36,37]. Gut microbiota dysbiosis leads to barrier dysfunction, allowing bacteria and LPS to enter the circulation. LPS binds to pattern-recognition receptors, activating macrophages to become M1-type. It complexes with Toll-like receptor 4, triggering the synthesis of pro-inflammatory factors like IL-1β, TNF-α, MMPs, and free radicals. This causes secondary inflammation in joint tissues and systemically, worsening osteoarthritis [38].

In summary, gut microbiota diversity is lower in osteoarthritis patients than in the healthy population. Gram-negative bacteria, along with *Streptococcus* spp., *Cholera* spp., and *Desulfovibrio* spp., may be the main causative organisms in osteoarthritis.

### 2.3. Gut Microbiota and Osteoporosis

Osteoporosis (OP), characterised by decreased bone mineral density and heightened fracture risk, shows differences in gut microbiota diversity between patients and healthy controls. In osteoporosis patients, the abundance of dominant gut microbiota is reduced, while that of opportunistic pathogenic bacteria increases, paralleled by decreased bone density [39,40]. Insulin-like growth factor 1 (IGF-1) stands as the pioneering hormone associated with the gut–bone axis. In an experiment involving the colonisation of germ-free (GF) mice, which inherently lack a gut microbiome, with the microbiota from specific pathogen-free (SPF) mice, a detailed analysis of bone formation processes showed a notable increase [41]. Furthermore, serum levels of IGF-1 substantially increased in colonised mice, with production reported in the liver and adipose tissue. In contrast, antibiotic treatment reducing the microbiome of conventional mice reported decreased IGF-1 production and less bone formation [41].

Research on OP, a condition with low bone density and high fracture risk, has explored the role of gut microbiota. In an ovariectomy-induced mouse OP model, broad-spectrum antibiotic treatment increased bone density and formation rate [42], but also altered gut microbiology, mainly affecting osteoclasts and enhancing bone resorption in post-pubertal mice [43]. In human OP patients, there is an increased abundance of pathogenic bacteria like *Actinomyces* spp. and *Escherichia coli*, which may trigger OP through inflammation [44]. Some bacteria in OP patients are correlated with bone resorption and formation markers. *Faecalibacterium* and *Dialister* abundance increases, while *Subdoligranulum* and *Blautia* abundance decreases in OP patients [45,46]. Gut microbiota dysbiosis causes barrier issues and over-production of harmful substances. These trigger inflammatory cytokines, activate the NF-κB pathway, and promote osteoclast differentiation. In OP, *Gram-negative bacteria*-secreted lipopolysaccharide causes inflammation, increases barrier permeability, and promotes osteoclast formation via the NF-κB pathway [47].

In summary, in OP patients, the abundance of dominant gut microbiota is reduced while that of opportunistic pathogens such as *Actinomyces*, *Escherichia coli*, *Actinomyces odontolyticus*, *Streptococcus sanguinis*, *Faecalibacterium*, *Dialister*, *Subdoligranulum*, and *Blautia* changes. Among them, these altered-abundance bacteria may be key causative agents in osteoporosis.

### 2.4. Gut Microbiota and Bone Metastasis

Tumour bone metastasis happens when malignant cells spread to the bone. The gut microbiota can indirectly affect this by influencing the tumour microenvironment and immune response [48]. In patients with tumour bone metastases, gut microbiota diversity is often reduced [49]. For example, in multiple myeloma (MM) patients, 16S rRNA gene sequencing shows lower alpha-diversity indices compared to healthy people. Tumours and their treatments may change the environment, leading to gut microbiota imbalance [50,51]. In a mouse model of breast cancer bone metastasis, following a period of antibiotic treatment, micro-computed tomography (micro-CT) revealed that the antibiotic- treated mice exhibited reduced bone destruction, a relatively intact trabecular structure, and a slower decline in bone density compared to the untreated group. A 4-week treatment of mice with breast cancer bone metastases using an antibiotic mixture led to significant improvements in the bone volume fraction and trabecular thickness of the tibia in the treated group versus the untreated group [52,53].

Studies have shown that in MM patients, compared to healthy controls, the composition of the gut microbiota changes significantly. There is a decrease in the number of *Actinobacteria phylum* bacteria and an increase in *Clostridium pumilus* and *Pseudomonas aeruginosa*, with the elevated level of *Clostridium pumilus* being associated with MM prognosis [50,54,55]. Another study revealed an increase in conditionally pathogenic bacteria in the gut of tumour bone-metastasis patients, such as significant enrichment of *Klebsiella* and *Streptococcus*, which can increase morbidity and mortality in immunocompromised populations. Thus, the increased abundance of *Klebsiella* pneumoniae has been associated with MM progression, suggesting a potential pathogenic role [49].

Alterations in the gut microbiota composition impact the bone marrow microenvironment, fuelling the development of MM. These changes modify numerous signals between the colonising gut microbiota and epithelial or immune cells, resulting in variations in inflammation, epithelial cell proliferation, or mucus secretion, and promoting cellular transformation or DNA damage [50,55,56]. The gut microbiota significantly impacts tumour development by disturbing microenvironmental homeostasis, modulating immune responses, and through the biofilms and toxic metabolites of gut bacteria. Analysing the composition of the gut microbiota could offer a diagnostic basis for evaluating tumour progression, particularly in the early stages [51,57]. Microbial communities within the tumour microenvironment either promote or inhibit malignancy development via diverse mechanisms. For instance, the faecal gut microbiota from colorectal cancer patients can induce tumour formation in germ-free mice and mice exposed to carcinogens [51,57,58].

In summary, our findings indicate that the alpha-diversity of the gut microbiota is significantly reduced in MM patients compared to healthy individuals, suggesting a potential link between gut microbiota dysbiosis and MM progression. *Actinobacteria phylum*, *Clostridium pumilus*, *Pseudomonas aeruginosa*, *Klebsiella*, and *Streptococcus* are likely key causative agents in multiple myeloma. Future studies should explore the underlying mechanisms and potential therapeutic interventions targeting the gut microbiota (Table 1).

## 3. Interaction Between Metabolites of Gut Microbiota with GPCRs

### 3.1. GPCRs as Key Receptors for Sensing Gut Microbiota Metabolites

#### 3.1.1. Response of GPR41, GPR43 and GPR109a to SCFAs

SCFAs (short-chain fatty acids), including acetic, propionic, and butyric acids, are products of dietary fibre fermentation by gut microbiota. They are energy sources and signalling molecules, and their role in bone metabolism has drawn much attention [72].

SCFAs activate GPCRs to influence bone metabolism, regulating osteoblasts and osteoclasts to balance bone formation and resorption, vital for bone density and osteoporosis prevention [14,73]. For instance, butyric acid may enhance osteoblast differentiation and suppress osteoclast activity, protecting against osteoporosis [14]. In vitro, it upregulates osteoblast markers like ALP [73], stimulates bone formation by increasing OPN (Osteopontin) and BSP production, and inhibits osteoclast differentiation by increasing OPG (Osteoprotegerin) production, having implications for osteoporosis treatment [73].

SCFAs are believed to promote osteoblast differentiation and inhibit osteoclast activity. Butyric acid has also been found to inhibit osteoclast activity. Sodium butyrate can decrease the phosphorylation level of the α/β subunit of NF-κB kinase and increase cytoplasmic subunit p65 content, thus inhibiting the activation of the NF-κB signalling pathway, which is central to various inflammatory and immune responses. This leads to a reduction in osteoclast production and activity, showing potential value in osteoporosis treatment [74].

GPR41 and GPR43 are two known specific short-chain fatty acid receptors. SCFAs act on GPR41 and GPR43, and their activation regulates energy expenditure, insulin sensitivity, and inflammatory responses. In bone metabolism, SCFAs promote osteoblast differentiation and inhibit osteoclast activity via GPR41 and GPR43 activation, potentially protecting against osteoporosis [14]. SCFAs promote osteoblast differentiation through the GPR41/43 signalling axis. For example, a study showed that in the presence of acetic acid, bone marrow-derived MSCs from wild-type mice differentiated into osteoblasts, leading to reduced mineralisation and downregulation of bone formation markers like Phex, Ptgs2, and Col1a1 [30], indicating that SCFAs regulate the osteogenic and lipogenic differentiation of MSCs through the GPR41/43 signalling axis, thus affecting bone mass.

The study of that *Akkermansia muciniphila* and its metabolite propionic acid regulate neuronal mitochondrial fission and autophagy through GPR41 and GPR43 receptors to maintain mitochondrial homeostasis, and it was found that the action of propionic acid on the GPR43 receptor promotes PINK1/PARKIN-mediated mitochondrial autophagy and maintains neuronal mitochondrial homeostasis [75]. In contrast, mitochondrial autophagy can promote osteoclastogenesis through the SIRT1, PINK1/Parkin, FOXO3, and PI3K signalling pathways [76].

Butyrate, a short-chain fatty acid, and niacin can bind to GPR109a as ligands, activating it and initiating downstream signalling pathways to regulate cellular functions [77,78]. A balanced gut microbiota aids in inducing and maintaining regulatory Tregs. Tregs secrete anti-inflammatory cytokines, which indirectly affect GPR109a expression and function, keeping it at normal levels for immunomodulation. Conversely, gut microbiota dysregulation triggers an inflammatory response, releasing many inflammatory factors that may inhibit GPR109a expression or disrupt its signalling [79]. GPR109a regulates the functions of osteoblasts and osteoclasts. It promotes bone formation by regulating the signalling pathway in osteoblasts, upregulating osteogenesis-related gene expression and enhancing osteoblast activity. Simultaneously, it inhibits the differentiation and maturation of osteoclast precursor cells, reducing osteoclast number and activity, and thus inhibits bone resorption to maintain bone metabolism balance. In osteoporosis, normal GPR109a function helps prevent excessive bone loss [80,81].

It has been shown that GPR109A deletion improves bone mass by inhibiting bone resorption, and measurements of bone remodelling markers showed that the expression of a bone resorption marker (histone enzyme K) was significantly lower in GPR109A-deficient mice, whereas the expression of β-catenin was higher than that in wild-type mice. β-catenin has an important role in bone metabolism, and its high expression may contribute to the maintenance of bone mass [81].

GPR109A controls the formation of NETs by regulating the ROS/PAD4/Cit-H3 signalling axis [82], whereas NETs enhance RANKL-induced TLR4- and TLR9-dependent osteoclastogenesis and increase osteoclast resorption in vitro [82]. GPR109a inhibits osteoclastogenesis by controlling NETs. The effects of hippuric acid (HA) and 3-(3-hydroxyphenyl)propionic acid (3-3-PPA) on the promotion of bone formation have also been investigated, whereas GPR109A plays an important role in osteoclast differentiation, and HA- and 3-3-PPA-mediated bone resorption mediates the inhibition of bone resorption during bone development [80].

**Figure 1:**
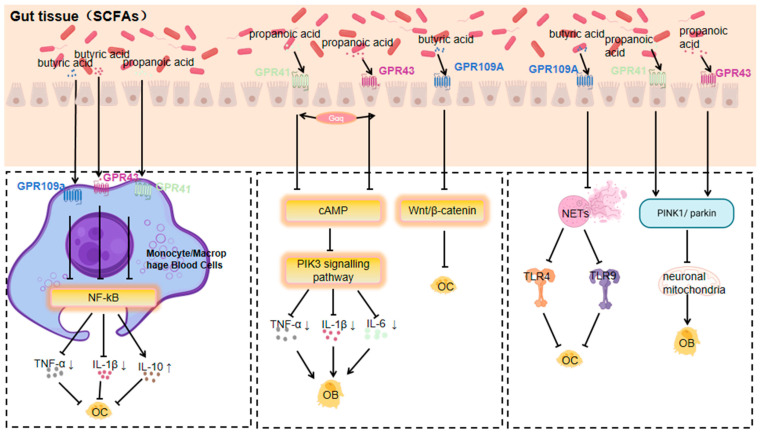
Mechanisms by which GPR41, GPR43, and GPR109a respond to short-chain fatty acids affecting orthopaedic diseases (Arrows indicate an increase or decrease in the impact factor).

SCFAs impact bone metabolism by activating GPCRs. Due to their effects on osteoblast and osteoclast function, SCFAs have a potential therapeutic role in orthopaedic diseases, especially osteoporosis, and thus have drawn significant attention. Researchers are exploring the modulation of gut microbiota to increase SCFA production as a novel therapeutic strategy [83] (Figure 1).

#### 3.1.2. TGR5 Response to Bile Acids

Bile acids, biologically active substances generated from cholesterol metabolism in the liver, participate in regulating multiple metabolic and inflammation-related physiological processes. They act via the activation of membrane receptor GPBAR/TGR5 and nuclear receptor FXR [84]. The gut microbiota, on the other hand, impacts bile acid metabolism and the composition of the bile acid pool by producing various enzymes like bile salt hydrolases and 7α-dehydroxylases. Using these enzymes, the gut microbiota can modify the bile acid pool composition through dehydrogenation, dehydroxylation, and desulphurisation, and then regulate bile acid metabolism by affecting bile acid receptors (such as farnesol X receptor) prior to feedback [85].

Bile acids influence energy metabolism and bone resorption by activating GPCRs like TGR5 and GPBAR1 (GPR35). TGR5, a primary bile acid sensor, is linked to various physiological processes, including energy expenditure and bone metabolism [86]. Research indicates that TGR5 activation promotes osteoblast differentiation and proliferation while inhibiting osteoclast activity, jointly contributing to increased bone density and enhanced bone quality [87].

TGR5, a member of the GPCR superfamily, serves as a receptor not only for bile acids but also for various selective synthetic agonists, regulating signalling pathways like NF-κB, AKT, and extracellular signal-regulated kinase (ERK) [88]. TGR5 activation is linked to enhanced bone density and quality, likely through promoting osteoblast differentiation and inhibiting osteoclast activity [87].

TGR5 is expressed in the osteoblast-like cell line MC3T3-E1 cells, and osteogenic medium (OM) stimulation boosts TGR5 expression. The specific TGR5 agonist GPBARA can increase ALP activity, matrix mineralisation, and the expression of osteoblast differentiation marker genes (such as ALP, OCN, and Osx) by promoting Runx-2 expression [89].

Tgr5−/− mice show some effects through enhanced leptin and Fgf21 signalling [90]; leptin acts as an anabolic bone factor in vivo, and the direct effects of leptin on osteoblasts may outweigh its effects via the central nervous system [91].

#### 3.1.3. GPR81 Response to Lactate

Lactate is produced by lactic acid bacteria in the gut microbiota using fermentable carbohydrates [92], and the presence of *bifidobacteria* and other beneficial bacteria and their activity have a significant effect on the production and utilisation of lactic acid by lactic acid bacteria [93]. Lactic acid produced by certain lactic acid bacteria can inhibit the growth of harmful bacteria such as *E. coli* and *Shigella*, while increasing the number of beneficial bacteria such as *bifidobacteria* and *lactobacilli* [92].

GPR81 mainly responds to lactate generated from the fermentation of gut microbiota, which is produced by lactic acid bacteria during carbohydrate metabolism [94]. In conditions like obesity or osteoarthritis, changes in the gut microbiota’s composition and function lead to an increase in lactate levels, either from adipose tissue release or bacterial metabolism in the joint’s local microenvironment, activating GPR81 [94]. Excess lactate activates GPR81 on adipocytes, regulating adipocytokine secretion such as leptin and lipocalin. This occurs by activating the downstream adenylyl cyclase–cAMP–protein kinase A (PKA) signalling pathway through G protein coupling [94]. High leptin levels boost osteoclast activity and bone resorption, while low lipocalin levels weaken its promotion of osteoblasts, disrupting the bone formation–resorption balance, leading to reduced bone mass and strength [95].

Lactate receptor GPR81 signalling in colonic dendritic cells and macrophages has been found to play an important role in colonic homeostasis [96]; GPR81 has also been found to play an important role in the regulation of osteoclast differentiation and function, and osteoclast-led bone resorption is an important part of the bone remodelling process and plays a key role in the regulation of bone mass [97].

At the start of osteoarthritis, the joint’s local microenvironment changes, and lactate from gut microbiota reaches the joints via blood circulation or local diffusion. Activation of GPR81 on articular chondrocytes and synoviocytes alleviates the inflammatory response by inhibiting the mitogen-activated protein kinase (MAPK) signalling pathway and reducing the release of inflammatory mediators like TNF-αand IL-1β.

Simultaneously, GPR81 activation can influence genes related to cartilage extracellular matrix synthesis by regulating relevant transcription factors. For example, it promotes type II collagen and proteoglycan synthesis, inhibits matrix metalloproteinases expression, and reduces extracellular matrix degradation, thus slowing down osteoarthritis progression [98,99].

#### 3.1.4. GPR84 Response to MCFAs

Medium-chain fatty acids (MCFAs) are a group of fatty acids containing 6–12 carbon atoms. In the gut microbiota of animals, medium-chain fatty acids are found mainly in the form of triglycerides [100]. Research suggests that the gut microbiota may be able to produce MCFAs directly; for example, microorganisms capable of producing MCFAs are present in the gut of insects [100]. They are unique in their digestion, absorption, transit, and oxidation in the animal body, and can play a role in rapid energy supply, improving fat deposition, inhibiting bacteria, and adjusting gut microbiota and structure [101,102,103,104].

MCFAs have certain antimicrobial effects, which can inhibit the growth of harmful bacteria in the tract and increase the number of beneficial bacteria, thus regulating the balance of gut microbiota [100]. MCFAs can promote the renewal and repair of epithelial cells and maintain the integrity of the mucosal barrier [100].

Certain anaerobic bacteria produce MCFAs during the metabolism of lipids and proteins, and MCFAs act as ligands for GPR84 [105]. MCFAs produced by gut microbiota activate GPR84 on bone marrow cells, likely through G protein-coupled activation of the phospholipase C (PLC)–inositol triphosphate (IP3)–calcium ion (Ca^2+^) signalling pathway. This activation promotes the differentiation of bone marrow mesenchymal stem cells into adipocytes, increasing bone marrow adipogenesis. Meanwhile, it inhibits the differentiation of these stem cells into osteoblasts, reducing osteoblast number and activity, and thus decreasing bone formation. Additionally, the increase in bone marrow adipocytes may secrete factors that suppress osteoblast function, further reducing bone mass and promoting osteoporosis development [106,107]. All authors provided critical curation on drafts and reviewed and edited the final manuscript.

**Figure 2:**
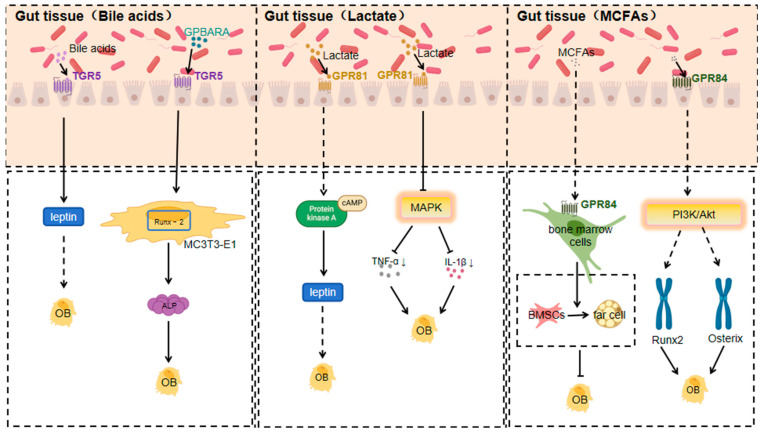
Mechanisms of action of TGR5, GPR81, and GPR84 in response to specific gut microbiota metabolites affecting orthopaedic diseases (Arrows indicate an increase or decrease in the impact factor).

It was found that CRC cells downregulated the expression of GPR84 in BMM to promote osteoclastogenesis in an IL-11-dependent manner, and therefore, GPR84 may be a potential therapeutic target to attenuate CRC metastasis-induced bone destruction [108] (Figure 2).

#### 3.1.5. Response of GPR91 to Succinate

Microorganisms produce various substances through metabolic activity, of which succinic acid is an important metabolite [109]. Many gut microorganisms produce succinic acid through specific metabolic pathways. For example, some strains of *E. coli* can produce succinic acid under anaerobic conditions using a mixture of sugars from non-food sources of soy hydrolysates [110]. Succinic acid produced by gut microbiota can positively affect host health by regulating the host’s metabolic processes [111]. And succinic acid has a novel role in regulating epithelial barrier function [112].

Multiple bacteria in the gut are involved in succinate production [113,114]. The succinate receptor GPR91, a GPCR, is expressed in diverse cell types and tissues. When macrophages expressing SUCNR1/GPR91 are activated by inflammatory signals, they change their metabolism, accumulate succinate, and release it into the extracellular environment [115]. Simultaneously, they upregulate GPR91 expression. GPR91, acting as an autocrine and paracrine sensor of extracellular succinate, enhances IL-1β production, which in turn affects osteoblasts. GPR91-deficient mice lack this metabolic sensor, showing reduced macrophage activation and IL-1β production during antigen-induced arthritis. Succinate is abundant in the synovial fluid of RA patients, and this fluid triggers macrophage IL-1β release in a GPR91-dependent way [116], which in turn affects osteoblasts.

Activation of GPR91 by succinic acid promotes the production of NLRP3, IL-1β, VEGF and MMP-13 in human peripheral blood mononuclear cells (PBMCs) and this process is achieved by increasing p65 phosphorylation and inhibiting the NF-κB signalling pathway, which is essential for the functional regulation of osteoblasts (osteoblasts, osteoclasts, etc.) in bone tissue [117].

In summary, a GPR91/succinate-dependent positive-feedback loop of macrophage activation is uncovered, and GPR91 antagonists are suggested as a novel therapeutic approach for treating orthopaedic diseases.

#### 3.1.6. GPR4 Response to Acids

Members of the gut microbiota, such as *Bifidobacteria*, actively participate in the fermentation process of dietary fibre in the normal environment, producing acids [118], such as short-chain fatty acids, which lower the pH value. GPR4, a pH-sensing receptor, plays an important role in the regulation of inflammatory and immune responses [119].

Gut microbiota play a role by being involved in the metabolic processes of phosphatidylcholine as well as by influencing the production of its hydrolysis products, LPC production and action [120]. GPR4 is a GPCR that can be activated by sphingosylphosphatidyl choline (SPC) and lysophosphatidyl choline (LPC) [121].

The proton-activated G protein-coupled receptor GPR4 regulates OA development by modulating the CXCL12/CXCR7 signalling pathway. Activated GPR4 impacts the CXCL12/CXCR7 pathway, crucial for chondrocyte proliferation, differentiation, and extracellular matrix metabolism. Over-activation or dysregulation of this pathway leads to abnormal chondrocyte function and accelerated cartilage degradation, offering new treatment ideas [122].

Another study found that the GPR4 antagonist NE 52-QQ57 inhibits the degradation of type II collagen in human chondrocytes induced by advanced glycosylation end-products (AGE). AGE accumulates in vivo, is closely related to OA progression, destroys type II collagen in chondrocytes, and damages the cartilage structure. GPR4 antagonists can block this process, suggesting GPR4’s key role in AGE-induced cartilage damage. Inhibiting GPR4 can reduce AGE-mediated destruction of the cartilage extracellular matrix, providing a potential pharmacological intervention for OA treatment [123].

#### 3.1.7. Response of GPR120 to Medium- and Long-Chain Unsaturated Fatty Acids

Gut microbes may synthesise and transform polyunsaturated fatty acids [124,125]. *Enterococci*, *Lactobacillus*, *Bifidobacterium* and other gut microbiota can convert unsaturated free fatty acids into medium- and long-chain fatty acids [126]. DHA- and EPA-rich polyunsaturated fatty acids can be obtained by consuming deep-sea fish and algae [127,128].

GPR120, a G protein-coupled receptor for medium- and long-chain unsaturated fatty acids like DHA and EPA, is activated by them. It is widely expressed in various tissues and cell types and has multiple functions in regulating systemic metabolism and inflammatory homeostasis [129].

Studies indicate that GPR120 is a key inflammatory regulator in OA development. GPR120 levels are lower in OA patients than in healthy controls. In an ACLT-induced OA animal model, GPR120 knockout mice had accelerated OA development, with more severe inflammation, cartilage degeneration, and subchondral bone abnormalities. Conversely, the GPR120 agonist docosahexaenoic acid (DHA) has an anti-inflammatory effect on human chondrocytes in vitro. In a skin defect model, DHA-activated GPR120 enhanced wound repair and reduced the number of CD68⁺ cells in mice. This implies that GPR120 activation may relieve OA symptoms by reducing inflammation, offering a potential target for OA treatment [130].

Regarding GPR120’s regulation of bone metabolism, it may influence bone metabolism by regulating the activities of bone cells such as bone marrow mesenchymal stem cells, osteoblasts, osteoclasts, and chondrocytes. Through specific signalling pathways, GPR120 can promote or inhibit the differentiation and proliferation of these cells to maintain bone metabolism balance. In orthopaedic diseases, a regulatory imbalance may cause disorders, and GPR120 is expected to be a crucial link in regulating bone metabolism and treating related diseases [131].

**Figure 3:**
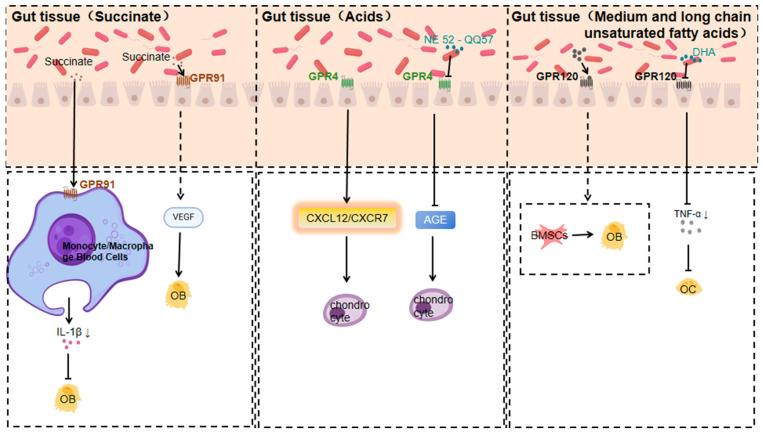
Mechanisms of action of GPR91, GPR4, and GPR120 in response to specific gut microbiota metabolites affecting orthopaedic diseases (Arrows indicate an increase or decrease in the impact factor).

DHA was found to inhibit inflammation-induced osteoclast formation and bone resorption via GPR120 in vivo. In vitro, DHA inhibited RANKL- and TNF-α-induced osteoclast formation. In vivo, DHA co-administered with LPS reduced the number of osteoclasts, the proportion of resorption pits, and related inflammatory factor levels. GPR120 antagonists or GPR120-deficient mice abolished DHA’s inhibitory effect. This suggests that GPR120 plays a significant role in regulating inflammation-associated bone resorption by inhibiting osteoclast formation and bone resorption, which is potentially relevant for preventing and treating orthopaedic disorders like inflammation-induced bone loss [132] (Figure 3).

#### 3.1.8. Response of GPR35 to Tryptophan Metabolites

Tryptophan metabolites, especially kynurenine, are generated from tryptophan through the action of gut microbiota and host enzymes. As an endogenous ligand for GPR35, kynurenine may impact bone metabolism and immune regulation by activating GPCRs like GPR35. The accumulation of kynurenine is linked to autoimmune diseases and can regulate the bone immune microenvironment by influencing the functions of T cells and dendritic cells [133].

GPR35 is an orphan GPCR, kynurenine, a tryptophan metabolite, possibly its endogenous ligand. It is highly expressed in a variety of immune cells and can regulate the release of inflammatory factors in a bi-directional and cell-type-dependent manner. For instance, kynurenine/GPR35 signalling decreases LPS-induced TNF-α release in human peripheral blood mononuclear cells and inhibits LPS + ATP-induced IL-1β secretion in THP-1 cells, highlighting the anti-inflammatory effects of GPR35 [134].

And gut microbes may synthesise lysine [135]. These metabolites, upon activating GPR35, can influence bone metabolism and immune regulation [136].

Kynurenine accumulation is linked to autoimmune diseases and can regulate the bone immune microenvironment by acting on T cell and dendritic cell functions [137]. Tryptophan metabolites are found to activate GPR35 and inhibit osteoclast differentiation, indicating GPR35’s potential role in bone resorption and formation [138].

GPR35 may negatively regulate osteoclast activity. It does so by inhibiting p65 phosphorylation and suppressing IκBα degradation. Since p65 is a key transcription factor in the NF-κB signalling pathway and its phosphorylation along with IκBα degradation are crucial for NF-κB activation, this reveals GPR35’s potential role in bone resorption [139,140].

Moreover, GPR35 activation may positively regulate osteoblast activity. It achieves this by strengthening the Wnt/β-catenin signalling pathway, a core pathway in osteoblast differentiation and bone formation, and upregulating RUNX2. RUNX2, an osteoblast-specific transcription factor, is essential for osteoblast differentiation and function [141].

#### 3.1.9. Response of GPR78 to Hydrogen Sulphide

Hydrogen sulphide, a gaseous signalling molecule, activates GPCRs and is related to functions like vasodilation, neurotransmission, and anti-inflammatory and antioxidant responses. It reduces inflammatory factor production via GPCR activation, having anti-inflammatory effects on osteoarthritis and osteoporosis. Gut microbiota’s sulphate-reducing bacteria (e.g., *Desulfovibrio* and *Desulfobulbus*) can convert dietary sulphur compounds to hydrogen sulphide anaerobically [142]. Through activating GPCRs like GPR78, hydrogen sulphide may influence the functions of osteoblasts and osteoclasts, thus affecting bone metabolism. The antioxidant and anti-inflammatory properties of hydrogen sulphide could be beneficial for treating osteoarthritis and osteoporosis [143].

Hydrogen sulphide’s effects on orthopaedic diseases mainly stem from its anti-inflammatory actions. It reduces the expression of inflammatory factors like IL-1β and TNF-α, showing potential therapeutic value for osteoarthritis, osteoporosis, and other orthopaedic conditions [142]. Additionally, hydrogen sulphide influences the onset and progression of osteoporosis by affecting bone cell metabolism and function, including the activities of osteoblasts and osteoclasts [144]. Moreover, it has strong antioxidant properties, capable of neutralising ROS and reactive nitrogen species (RNS) to mitigate oxidative-stress-induced damage to bone cells. This antioxidant effect can protect bone cells from oxidative harm and maintain bone health [145].

Gut microbiota can promote hydrogen sulphide production [146]. GPR78, an orphan G protein-coupled receptor, is expressed in diverse cell types, including immune and gastro cells [136].

Hydrogen sulphide may impact the functions of osteoblasts and osteoclasts by activating GPCRs like GPR78, thereby affecting bone metabolism. The antioxidant and anti-inflammatory properties of hydrogen sulphide could be beneficial for treating osteoarthritis and osteoporosis [147].

**Figure 4:**
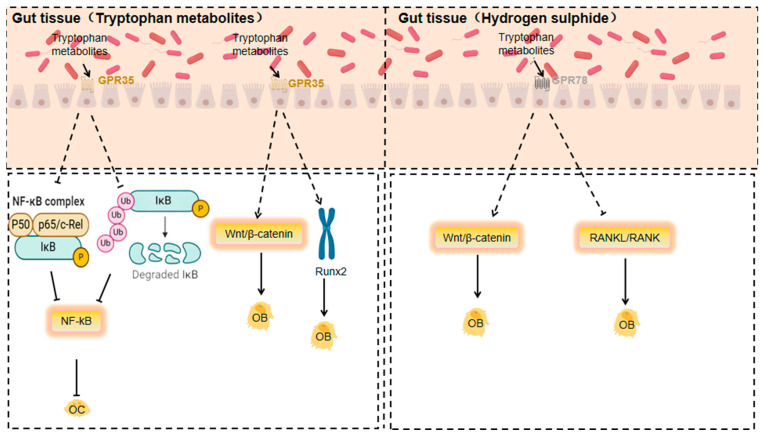
Mechanisms of action of GPR35 and GPR78 in response to specific gut microbiota metabolites affecting orthopaedic diseases (Dashed lines indicate that the indicated connection, process or trend is based on speculation, assumptions or is not yet fully substantiated).

In conclusion, gut microbiota metabolites are crucial in orthopaedic diseases via GPCR activation. These interactions offer potential targets for developing new therapeutic strategies. Future research should further explore the specific mechanisms between these metabolites and GPCRs to better understand their roles in orthopaedic diseases and devise more effective and personalised treatment methods (Figure 4).

## 4. Role of Metabolites and GPCRs in Orthopaedic Diseases and Molecular Mechanisms

### Signal Transduction Pathways and Their Effects on Orthopaedic Diseases

In orthopaedic diseases, various signal transduction pathways play crucial roles. The cAMP-PKA signalling pathway is involved when GPR41, GPR43, and TGR5 are activated. The dissociation of the G protein α subunit (Gαs) from GDP and its binding to GTP activates adenylyl cyclase (AC), raising the cAMP level, which in turn activates PKA. PKA acts on downstream target proteins like CREB and regulates the functions of osteoblasts and osteoclasts, influencing bone resorption and formation [148,149,150]. Meanwhile, the PI3K-Akt signalling pathway, activated by the G protein α subunit (Gαi/o) upon GPCR activation, is essential for cell survival, proliferation, and metabolism, and it also regulates osteoblast differentiation and bone resorption [151,152]. GPCR activation can trigger MAPK signalling pathways (ERK, JNK, p38 MAPK) via Ras. These pathways are vital for cell processes such as proliferation, differentiation, and apoptosis, and they regulate osteoblast and osteoclast functions, affecting bone resorption and formation [153,154]. The activation of GPCRs accelerates fracture healing through the cAMP-PKA signalling pathway, which promotes angiogenesis and osteoblast proliferation in the fracture region. The activation of the PI3K-Akt signalling pathway also promotes osteoblast differentiation and bone matrix synthesis, both important for fracture repair [155,156]. Gut microbiota metabolites, by activating GPCRs, trigger signal transduction pathways crucial for orthopaedic diseases. These pathways impact osteoblast function and bone metabolism, offering new treatment targets. Future research should focus on clarifying their specific mechanisms to develop more effective treatment strategies.

## 5. Mechanisms of GPCR Regulation of Gut Microbiota

### 5.1. Immune Regulation

GPCRs are crucial in regulating the immune response and thus affect the balance of the gut microbiota [83].

Gut immune activity was found to inhibit the developmental promotion of Drosophila by Acinetobacter, reflecting the interaction between the gut microbiota and host immunity in influencing host development [157].

Butyric acid, an SCFA, can attenuate the inflammatory response by activating the GPR43 receptor, which may impact osteoarthritis development. Gut microbiota-produced SCFAs like acetic, propionic, and butyric acids can activate GPR41 and GPR43 expressed on immune and epithelial cells [83]. Moreover, gut microbiota metabolites can influence the function of local T and B cells in joints, modulating the production of inflammatory factors such as TNF–α and interleukin 6 (IL-6), which are key in the osteoarthritis pathological process [37].

Research shows that SCFAs activating GPR43 enhance the phagocytic activity of neutrophils and macrophages, improving host defence against pathogens. This enhanced immune function helps maintain gut microbiota balance by preventing harmful bacteria overgrowth. Additionally, GPR43 activation promotes the production of anti-inflammatory cytokines like interleukin-10 (IL-10), which helps maintain immune homeostasis [14,158].

It was found that when the GPR35 gene was deleted, there was an increase in *Mycobacterium anisopliae* in GPR35-deficient mice, suggesting that GPR35 has an important role in the regulation of gut microbiota signalling [159].

It has also been shown that GPCRs are involved in the effects of γ-ketoc (γ-keto-compound) on dendritic cells in in vitro experiments using GPR40/120 agonists, suggesting that the GPCR signalling pathway is involved in the immunomodulation of bacterial metabolites [160].

### 5.2. Regulation of Bacterial Homeostasis by GPCRs

GPCR signalling pathways are affected by gut microbiota metabolites and can act on the gut microbiota. Activation of certain GPCR pathways impacts peristaltic and secretory functions, altering the microenvironment and influencing gut microbiota growth and colonisation. In orthopaedic conditions like osteoporosis caused by long-term glucocorticoid therapy, drug-induced interference with GPCR signalling can dysregulate the gut microbiota, which then worsens abnormal bone metabolism, forming a vicious cycle [161].

## 6. Research Progress of GPCR Activation in Orthopaedic Diseases

### 6.1. Drug Development

At present, the drug research and development for orthopaedic diseases is booming. A large number of drugs have been put into market applications. Meanwhile, a considerable number of drugs are under in-depth research. In addition, many drugs with potential therapeutic effects are also being actively explored and are expected to become effective means for treating orthopaedic diseases in the future. However, it should be noted that the side effects of many orthopaedic drugs can cause great harm to the human body (Table 2).

### 6.2. Exercise Regulates Gut Microbiota and Thus Activates GPCR

Moderate exercise can reshape the gut microbiota, increasing beneficial bacteria, reducing harmful ones, and improving metabolic efficiency. Aerobic exercise boosts faecal SCFA concentration, lowering colonic lumen pH and affecting the gut microbiota. SCFAs act on mucosal GPCRs like GPR43, with anti-inflammatory effects crucial for mucosal immune balance [171].

Alterations in gut microbiota, especially in arginine metabolism, influence skeletal mechanical adaptation. Variations in gut microbiota composition underlie the heterogeneity in skeletal mechanical responsiveness, with Trichoderma (Lachnospiraceae) playing a role. Administering Lachnospiraceae increases L-citrulline (2-amino-5-ureidovaleric acid) and L-arginine (2-amino 5-guanidinovaleric acid) expression in receptors, enhancing skeletal mechanical responsiveness [156] (Table 3).

### 6.3. Dietary Modulation of Gut Microbiota and Thus Activation of GPCR

Dietary intervention can impact the gut microbiota’s composition and function, thereby modulating GPCR signalling. For instance, dietary fibre promotes the growth of beneficial gut bacteria that produce SCFAs. SCFAs activate GPR41 and GPR43, which regulate energy metabolism and immune responses. A clinical trial by Slavin et al. [177] showed that increased dietary fibre intake altered the gut microbiota composition and improved metabolic parameters, and these changes were linked to increased SCFA production and GPCR activation (Table 4).

## 7. Future Research Directions

### 7.1. New GPCR Discovery

With the development of high-throughput screening, genomics, and proteomics, the discovery of new GPCRs has sped up. These new receptors could be involved in unknown signalling pathways and offer new treatment targets for orthopaedic diseases [149,188].

New GPCRs may directly regulate bone metabolism, like influencing osteoporosis development by modulating the balance of bone formation and resorption. In inflammatory bone diseases such as osteoarthritis, they may be involved in releasing inflammatory mediators, activating immune cells, and modulating the inflammatory response to reduce joint inflammation and pain [189,190].

They might also play a role in pain signalling, especially in neuropathic and injury-sensitive pain, providing new pain management strategies. During fracture healing, new GPCRs may regulate angiogenesis, promoting blood supply to the fracture site and accelerating the repair process [149].

### 7.2. Strategies for Modulating the Gut Microbiota

Faecal microbiota transplantation (FMT) is an emerging method. It transfers faecal material from a healthy donor to a recipient to restore a healthy gut microbiota. A study by Vrieze et al. [191] found that FMT from a lean donor to a patient with metabolic syndrome changed the gut microbiota composition. FMT may modulate GPCR activity by altering the gut microbial environment and metabolite production.

### 7.3. Personalised Therapeutic Strategies

In orthopaedic diseases, the individual differences in gut microbiota play a pivotal role. GPCRs functioning as sensors for gut microbiota metabolites offer significant potential for personalised treatment strategies. Gut microbiota-derived metabolites, including SCFAs, bile acids, and TMAO, activate specific GPCRs, thereby influencing bone metabolism and immune responses. However, the distinct composition and function of gut microbiota among individuals lead to varied responses to GPCR activators or antagonists. For instance, the inter-individual variation in SCFA levels can directly impact the efficacy of GPR41 and GPR43 agonists [72].

Analysing the composition of the gut microbiota and the levels of related metabolites can serve as a predictive tool for patient responses to GPCR-targeted drugs, facilitating the implementation of precision medicine. Patients with a high production of SCFAs may exhibit a more favourable response to GPR41 and GPR43 agonists. Incorporating these individual differences into drug development can significantly enhance therapeutic efficacy while minimising adverse effects. An example of this approach is adjusting the dosages of TGR5-targeted drugs according to an individual’s bile acid metabolism [14,192].

Moreover, personalised diet and exercise can synergize with GPCR-based drug therapies to enhance their effectiveness. For example, increasing dietary fibre intake can promote the production of SCFAs, thereby augmenting the effects of GPR41 and GPR43 agonists [10]. This integrated approach combines the modulation of the gut microbiota through lifestyle changes with targeted drug interventions.

In conclusion, the development of personalised exercise and dietary treatment strategies for patients with different orthopaedic diseases is of great significance. Due to the differences in the gut microbiota of different patients, targeted regulation of the gut microbiota can affect its metabolites, which can activate different GPCRs and ultimately achieve the goal of improving orthopaedic diseases.

## 8. Conclusions

Exercise and diet modulate the gut microbiota, and gut microbiota and GPCR play an important role in the regulation of orthopaedic diseases. In the gut of many patients with orthopaedic diseases, the diversity, composition, and function of gut microbiota are altered, which can trigger inflammation, affect bone metabolism and immune homeostasis, and promote disease progression. Some metabolites of the gut microbiota activate GPCRs, which regulate bone metabolism and immune responses and maintain homeostatic regulation of the gut flora. Drug development of GPCRs for orthopaedic diseases has progressed with clinical applications and the emergence of new candidate molecules, but some of the drugs produce adverse effects, affect the immune system, and cause inflammation. Our individualised therapeutic strategy based on GPCR, which analyses the composition of the gut microbiota and metabolite levels based on individual differences in gut microbes, is expected to enable precision medicine through different diets and exercise.

In view of the current research status, the following research perspectives are proposed:

(1) In terms of the discovery and function of new GPCRs, the discovery of new GPCRs is expected to provide new avenues for personalised treatment of orthopaedic diseases.
(2) Moderate exercise and dietary interventions affecting the activity of GPCRs through the regulation of gut microbiota are expected to provide new protocols and strategies for personalised treatment of orthopaedic diseases.

## Figures and Tables

**Table 1:** Changes in gut microbial composition in patients and animals with orthopaedic diseases (‘↑’ represents an increase in the relative abundance of gut microbes. ‘↓’ represents a decrease in the relative abundance of gut microbes).

Type	Subject	Disease Model	Methods	Sample Size (n)	Changes in Gut Microbiota (Beneficial Bacteria)	Changes in Gut Microbiota (Pathogenic Bacteria)	Reference
RA	human	Rheumatoid arthritis patients	16S rRNA sequencing	52	*Fusicatenibacter* ↓ *Enterococcium* ↓	*Escherichia* ↑ *Eisenbergiella* ↑ *Klebsiella* ↑	[59]
RA	animal	Collagen-induced arthritis (CIA) rats	16S rRNA sequencing	around 10–20 per group	*Bifidobacterium* ↓ *Lactobacillus* ↓ *Pseudomonas fragilis* ↓	*Fusobacterium nucleatum* ↑ *Streptococcus* ↑ *Escherichia coli* ↑ *Prevotella* ↑ *Bacteroidetes* ↑	[60,61,62,63,64]
OA	human	Osteoarthritis patients	Metagenomic sequencing	89	*Agathobacter* ↓ *Ruminococcus* ↓ *Roseburia* ↓ *Subdoligranulum* ↓ *Lactobacillus* ↓	*Prevotella_7* ↑ *Clostridium* ↑ *Flavonifractor* ↑ *Klebsiella* ↑	[63,65]
OA	animal	Mono-iodine-acetic acid (MIA) induced OA animal models	Metagenomic sequencing or 16S rRNA sequencing	around 10–20 per group or 93	*Dictyostelium parvum* ↓ *Ackermannia* ↓ *Lactobacillus* ↓ *Bifidobacterium bifidum* ↓	*Enterobacteriaceae* ↑ *Staphylococcus* *Proteus* ↑ *Clostridium* ↑	[66,67]
OP	human	Osteoporosis patients	16S rRNA sequencing	2033	*Firmicutes* ↓ *Blautia* ↓ *Alistipes* ↓ *Megamonas* ↓ *Anaerostipes* ↓	*Lactobacillus* ↑ *Ruminococcus* ↑ *Bacteroides of Bacteroidetes* ↑	[46]
OP	animal	Ovariectomized (OVX) rats	16S rRNA sequencing	around 10–20 per group	*Bifidobacterium* ↓ *Lactobacillus* ↓ *Akkermansia muciniphila* ↓	*Streptococcus sanguinis* ↑ *Streptococcus gordonii* ↑ *Actinomyces odontolyticus* ↑ *Actinomyces graevenitzii* ↑ *Escherichia coli* ↑ *Enterococcus* ↑ *Bacteroides* ↑	[68,69]
BM	human	Tumour bone metastasis patients	Metagenomic DNA sequencing	79	*Megamonas* ↓ *Clostridia* ↓ *Akkermansia* ↓ *Gemmiger* ↓ *Paraprevotella* ↓	*Lactobacillales* ↑ *Bacilli* ↑ *Veillonella* ↑ *Streptococcus* ↑ *Campylobacter* ↑ *Epsilonproteobacteria* ↑ *Acinetobacter* ↑ *Pseudomonadales* ↑ *Moraxellaceae* ↑	[70]
BM	animal	Inoculation of tumour cells into animal bones	Metagenomic DNA sequencing or 16S rRNA sequencing	around 10–20 per group	*Bifidobacterium* ↓ *Lactobacillus* ↓ *Akkermansia muciniphila* ↓	*Fusobacterium nucleatum* ↑ *Escherichia coli* ↑ *Enterococcus faecalis* ↑ *Fusobacterium* ↑	[49,71]

**Table 2:** Information sheet on GPCR drug development progress for orthopaedic diseases.

Drug Development	Name of Drug	Mechanism of Action	Side Effects	Reference
Existing Drugs	Bisphosphonates	Used in the treatment of osteoporosis to increase bone density by activating the TGR5	Studies have found that phosphonates are associated with an increased risk of atypical femur fractures, bisphosphonates have been associated with an increased risk of osteonecrosis of the jaw (ONJ), particularly in patients undergoing dental surgery, and oral bisphosphonates may cause oesophagitis and other gastro symptoms.	[162,163,164,165]
	Denosumab	A monoclonal antibody against RANKL used for the treatment of osteoporosis and bone metastases that reduces bone resorption by inhibiting the RANKL-RANK signalling pathway	Studies have found that Denosumab may affect the immune system and increase the risk of infection. Denosumab treatment may cause hypocalcaemia, especially in patients with vitamin D deficiency.	[166,167]
	Prostaglandin	Used in the treatment of osteoarthritis to reduce inflammation and pain by activating EP2 and EP4 receptors	PGE2 analogues can cause gastro disturbances, increase the risk of cardiovascular events (especially in heart disease patients), and negatively impact renal function (especially in the elderly or those with kidney disease history).	[168,169]
New Drug Candidate Molecules	GPR41 and GPR43 Agonists	Activators of short-chain fatty acid receptors being investigated to promote osteoblast differentiation and inhibit osteoclast activity for the treatment of osteoporosis		[14]
	TGR5 Agonists	Activators of the bile acid receptor in development to improve energy metabolism and bone density		[87]
	AhR Agonists	Activators of the aromatic hydrocarbon receptor, potentially used in the treatment of osteoarthritis by modulating immune response and inflammation		[170]

**Table 3:** The effect of different exercises on gut microbiota.

Type	Changes in Gut Microbiota	Reference
High-intensity interval training	In bodybuilders, the genera *Clostridium*, *Eisenbergiella*, *Faecalibacterium*, *Haemophilus*, and *Sutterella* were more abundant, while *Bifidobacterium* and *Parasutterella* were less abundant. Probiotic species including *Bifidobacterium adolescentis*, *Bifidobacterium longum*, *Latilactobacillus sakei*, and SCFA-producing microorganisms (*Blautia wexlerae*, *Eubacterium hallii*) were less abundant in bodybuilders compared to controls.	[172]
Endurance training	At the phylum level, *Lentisphaerae* and *Acidobacteria* were detected after running, with their functions in the human gut remaining unknown. At the family level, there was an increase in *Coriobacteriaceae* and *Succinivibrionaceae*, where *Coriobacteriaceae* are involved in bile salts and steroid hormones metabolism and activation of dietary polyphenols. At the genus level, half marathon running led to a decrease in the levels of *Ezakiella*, *Romboutsia*, and *Actinobacillus*, and an increase in *Coprococcus* and *Ruminococcus bicirculans*.	[173]
Aerobic exercise	In a study by Durk et al. [174] on young healthy individuals, a higher *Firmicutes*/*Bacteroidetes* ratio was significantly correlated with higher VO2max. In a study on premenopausal women, participants with low VO2max had lower *Bacteroides* levels but higher *Eubacterium rectale* and *Clostridium coccoides* levels compared to those with high VO2max. A 12-week aerobic exercise training led to an increase in the relative abundance of intestinal *Bacteroides* and an improvement in cardiorespiratory fitness.	[174,175,176]

**Table 4:** The effect of different diets on gut microbiota.

Type	Changes in Gut Microbiota	Reference
Western Diet	This type of diet leads to a decrease in the abundance of several beneficial bacterial species, including *Akkermansia muciniphila*, *Faecalibacterium prausnitzii*, *Roseburia* spp., *Eubacterium* spp., and the bacteria in *Clostridium* cluster XIVa and IV.	[178,179]
Ketogenic Diet	In obese patients, the *Bacteroidetes*/*Firmicutes* ratio was significantly altered. After a very-low-calorie ketogenic diet (VLCKD), there was a reduction in *Eubacterium rectale* and *Roseburia*, while the abundance of *Akkermansia* and *Christensenellaceae* increased.	[180,181]
Vegan Diet	High intakes of indigestible carbohydrates (wheat bran and whole grain) typically lead to an increase in *Lactobacillus* spp. and *Bifidobacterium* spp. Whole grain barley and resistant starch are associated with an increase in *Ruminococcus* spp., *Eubacterium rectale*, and *Roseburia* spp., and a decrease in some Firmicutes phylum taxa such as *Clostridium* and *Enterococcus* species.	[182,183]
Gluten-Free Diet	In healthy volunteers, one month of a gluten-free diet (GFD) led to the following microbiota changes: a decrease in the populations of *Lactobacillus*, *Bifidobacterium*, and Roseburia; an increase in *E. coli*, *Enterobacteriaceae*, *Victivallaceae*, and *Clostridiaceae.*	[184,185]
Mediterranean Diet	In a study of twenty obese men, a one-year Mediterranean diet (MD) led to a decrease in *Prevotella* and an increase in *Oscillospira* and *Roseburia.* A long-term MD resulted in an increase in the relative abundance of *Parabacteroides distasonis*. A two-year MD had a positive effect, increasing the levels of *Bacteroides*, *Prevotella*, and some saccharolytic genera such as *Roseburia*, *Faecalibacterium*, and *Ruminococcus*.	[186,187]

## Abbreviations

AGE	advanced glycosylation end
CIA	collagen-induced arthritis
DHA	docosahexaenoic acid
ERK	extracellular signal-regulated kinase
FMT	faecal microbiota transplantation
GPCRs	G protein-coupled receptors
HA	hippuric acid
IGF-1	insulin-like growth factor 1
IL-6	interleukin 6
IL-10	interleukin-10
LPC	lysophosphatidyl choline
MAPK	mitogen-activated protein kinase
MCFAs	medium-chain fatty acids
MMPs	matrix metalloproteinases
MM	multiple myeloma
micro-CT	micro-computed tomography
MSCs	mesenchymal stem cells
OA	osteoarthritis
OM	osteogenic medium
OP	osteoporosis
OPN	Osteopontin
OPG	Osteoprotegerin
PI3K	phosphatidylinositol 3 kinase
PLC	phospholipase C
PGE2	Prostaglandin E2
RA	rheumatoid arthritis
RNS	reactive nitrogen species
SCFAs	short-chain fatty acids
SPC	phingosylphosphatidyl choline
SPF	specific pathogen-free
Tregs	T cells
TMAO	Trimethylamine *N*-oxide
